# A Comparative Study on COVID-19 Dynamics: Mathematical Modeling, Predictions, and Resource Allocation Strategies in Romania, Italy, and Switzerland

**DOI:** 10.3390/bioengineering12090991

**Published:** 2025-09-18

**Authors:** Cristina-Maria Stăncioi, Iulia Adina Ștefan, Violeta Briciu, Vlad Mureșan, Iulia Clitan, Mihail Abrudean, Mihaela-Ligia Ungureșan, Radu Miron, Ecaterina Stativă, Roxana Carmen Cordoș, Adriana Topan, Ioana Nanu

**Affiliations:** 1 Automation Department, Technical University of Cluj-Napoca, 400114 Cluj-Napoca, Romania; vlad.muresan@aut.utcluj.ro (V.M.); iulia.clitan@aut.utcluj.ro (I.C.); mihail.abrudean@aut.utcluj.ro (M.A.); mihaela.unguresan@chem.utcluj.ro (M.-L.U.); radu.miron@aut.utcluj.ro (R.M.); 2 Department of Infectious Diseases and Epidemiology, Iuliu Hațieganu University of Medicine and Pharmacy, 400347 Cluj-Napoca, Romania; briciu.tincuta@umfcluj.ro (V.B.); topan.adriana@umfcluj.ro (A.T.); 3 The Clinical Hospital of Infectious Diseases, 400003 Cluj-Napoca, Romania; 4 Alessandrescu Rusescu National Institute for Mother and Child Health, 020395 Bucharest, Romania; ecaterina.stativa@gmail.com (E.S.); ioana.nanu@insmc.ro (I.N.); 5 Robotics and Production Management Department, Technical University of Cluj-Napoca, 400114 Cluj-Napoca, Romania; roxana.cordos@mis.utcluj.ro

**Keywords:** COVID-19, SARS-CoV-2 virus, control design, prediction, pandemic dynamics, mathematical model, data processing, neural networks

## Abstract

This research provides valuable insights into the application of mathematical modeling to real-world scenarios, as exemplified by the COVID-19 pandemic. After data collection, the preparation stage included exploratory analysis, standardization and normalization, computation, and validation. A mathematical model initially developed for COVID-19 dynamics in Romania was subsequently applied to data from Italy and Switzerland during the same time interval. The model is structured as a multiple-input single-output (MISO) system, where the inputs underwent a neural network-based training stage to address inconsistencies in the acquired data. In parallel, an ARMAX model was employed to capture the stochastic nature of the epidemic process. Results demonstrate that the Romanian-based model generalized effectively across the three countries, achieving a strong predictive accuracy (forecast accuracy > 98.59%). Importantly, the model maintained robust performance despite significant cross-country differences in testing strategies, policy measures, timing of initial cases, and imported infections. This work contributes a novel perspective by showing that a unified data-driven modeling framework can be transferable across heterogeneous contexts. More broadly, it underscores the potential of integrating mathematical modeling with predictive analytics to support evidence-based decision-making and strengthen preparedness for future global health crises.

## 1. Introduction

The pandemic caused by COVID-19 has had a significant influence on Romania, significantly affecting a variety of public health sectors, healthcare infrastructure, and the overall well-being of the society.

The early reaction that Romania provided to the COVID-19 epidemic was defined by both accomplishments and problems of the country. Constraints in the healthcare system and social factors posed specific challenges to the country in its efforts to control the spread of the virus [1]. As the result of Romania’s considerable diaspora, one of the most serious challenges was the repatriation of a huge number of people from badly afflicted regions, mainly from Italy [2]. On the other hand, the prompt application of control measures was effective in preventing an increase in COVID-19 cases, which helped delay the overload of an already struggling healthcare system in the early stages of the epidemic [3,4]. For the purpose of facilitating early control measures, effective communication channels that touched all sectors of the society, from ordinary residents to governmental officials and high-ranking religious leaders, were used [5,6]. The lessons that may be learned from this experience highlight the need for a fast response that takes into account sociocultural factors during an epidemic [1].

The early spread of COVID-19 in Romania was largely impacted by imported cases from Italy and subsequent human-to-human transmission networks. These factors were responsible for the most significant impact. Nearly all of the administrative counties had a significant number of index cases that were mostly Romanians returning from Italy [7]. Of the first 147 patients to be recorded, 60% were imported cases. Of these, sixty-four came from Italy and twenty-four came from other countries [1,8]. Early local transmission networks were characterized by short routes (with a maximum of three transmission chains), restricted geographical dispersion, and a high degree of centralization, which indicated the existence of super-spreaders. These characteristics were brought about by successive importations within the same region [9]. According to the findings of the research, local transmission cycles were preceded by imported cases, the majority of which originated from Italy. This further emphasizes the influence that human transportation corridors have on the spread of the illness [2]. During the fourth wave of the COVID-19 pandemic, Romania was hit by a devastating outbreak that was marked by a fast increase in the number of infections and high fatality rates. Midway through the month of October 2021, the nation has reported close to 20,000 daily illnesses, with more than 500 fatalities occurring each day [10]. Despite the fact that vaccines were readily available, vaccination coverage remained low; by the time the fourth wave arrived, only around thirty percent of the population had been completely immunized. This scenario was brought about by a number of circumstances, including economic and social difficulties, inadequate support for the vaccination effort, political instability, and the dissemination of false information [11,12]. Vaccination attempts were further hindered by the absence of systematic discussions with significant social groups and the lack of confidence in state officials due to the absence of such consultations [13]. This circumstance highlights the important need to address vaccine reluctance and equip the healthcare system to properly react to national catastrophes. Vaccine hesitancy is a problem that has to be addressed [14].

In the case of Italy, two Chinese tourists who were visiting Rome tested positive for COVID-19 on 31 January 2020, which was the day that Italy disclosed its first confirmed instances of the virus [15]. However, it was not until the second half of February that the virus started to spread extensively in the northern regions of Lombardy and Veneto, notably in the vicinity of the city of Codogno, which became one of the first significant epicenters in Europe [16]. Italy started to see an exponential surge in the number of COVID-19 cases in the middle of February 2020, particularly in the region of Lombardy, where the virus spread swiftly. As a result of the initial epidemic cluster in Lombardy becoming a hot zone, infections began to spread into nearby areas [17]. Because of the large increase in the number of instances that occurred in Italy by March 2020, the country’s healthcare system, especially in the region of Lombardy, became overwhelmed [18,19]. Around the second and third weeks of March, when the nation’s healthcare system was under an incredible amount of strain, the country hit its highest point in terms of the number of new cases and deceased individuals [20]. As a result of the overwhelming number of COVID-19 patients, the healthcare system in Italy, particularly in the area of Lombardy, failed to properly manage the situation. The number of patients in intensive care units (ICUs) approached their maximum capacity, and hospitals were overcrowded [21,22]. As a result of insufficient resources, particularly ventilators and intensive care unit beds, medical professionals were forced to make difficult choices about the patients who would receive treatment [23]. There was a major lack of medical supplies, especially personal protective equipment (PPE) for those who work in the healthcare industry. When it came to addressing these shortages, Italy was forced to depend on the assistance and contributions of other countries. Because of this, those who worked in healthcare were exposed to a greater risk of infection [24].

In spite of the fact that it often offered high-quality medical treatment, Italy’s healthcare system was not adequately equipped to deal with a pandemic of this magnitude [25]. The unexpected increase in the number of cases, particularly in Lombardy, brought to light deficiencies in the system’s capacity to react in a prompt and effective manner. The Italian government decided to implement a partial lockdown in the area of Lombardy on 21 February 2020. This was because the epidemic was particularly acute in that region [26].

On the other hand, by March 9th, the government had extended the lockdown over the whole country and had implemented tight precautions across the entire nation in an effort to prevent the virus from spreading further. This included the closure of schools and companies that were not necessary, as well as restrictions on travel [27]. The Italian government implemented stringent social distancing measures, which included the limitations on public meetings, the closing of public areas, and the prohibition of large-scale events [28]. Citizens were strongly encouraged to remain indoors if at all possible. These steps were essential in the effort to restrict the spread of the virus; nevertheless, the fast spread of the virus in specific regions posed a challenge to the efficiency of these measures. Although Lombardy was the region that was negatively affected the most, the virus quickly spread to other regions, notably the northern region [29]. Despite the fact that the government concentrated its efforts on regional containment techniques, it was forced to adopt lockdowns throughout the country as the situation became progressively worse [30].

When the COVID-19 epidemic reached Switzerland’s borders in the early 2020, the country, like the majority of other nations, was confronted with enormous obstacles [31]. The reaction of the country, which was formed by its highly decentralized healthcare system, robust public health infrastructure, and strong emphasis on individual responsibility, played an important part in the management of the early waves of the infection [32]. The canton of Ticino, which is situated close to the border with Italy, was the location where the first verified case of COVID-19 in Switzerland was reported on 25 February 2020. The patient, a man in his seventies, had flown to Milan, Italy, which was one of the first places in Europe to be severely affected by the widespread spread of the virus [33]. The Swiss Federal Office of Public Health (FOPH) quickly established that the virus was spreading locally, especially in places that have strong relations to Italy, such as Geneva and Ticino. This was particularly problematic for the region [34]. Measures that were intended to prevent the spread of the virus were among the early responses that Switzerland took in reaction to the epidemic. During the exceptional circumstance that was announced by the Swiss Federal Council on 16 March 2020, lockdowns were implemented over the whole country, and schools, restaurants, and other non-essential enterprises were shut down [35]. Citizens were strongly encouraged to maintain a social distance from one another, and large public gatherings were prohibited [36].

In March of 2020, Switzerland was hit by its first wave, which was characterized by an increase in infection rates. A considerable rise in the number of cases was seen throughout the nation, with the number of daily new infections surpassing 1000 by the middle of March. With a rise in the number of COVID-19 patients who were hospitalized, the healthcare system, and notably intensive care units (ICUs), came under increasing amounts of strain [37]. The transmission rate, on the other hand, started to decrease by the end of April and into May as a result of the swift adoption of stringent public health measures, which led to a progressive reopening of the economy. Additionally, the pandemic had a considerable influence on the economy of Switzerland. During the year 2020, the economy of the nation had a contraction of 2.4%, with the tourist and hospitality industries suffering the most severe consequences. There was a wide variety of economic relief measures that were implemented by the Swiss government [38]. These measures included financial assistance for companies, workers, and self-employed people who were impacted by the epidemic. It was possible for companies to keep their workers even while the economy was in a slump because of the use of measures such as short-time employment, which prevented widespread layoffs [39]. The durability and flexibility of Switzerland’s healthcare system is shown by the country’s response to the COVID-19 epidemic. The nation successfully deployed public health measures, including lockdowns, vaccination drives, and economic relief programs, in order to reduce the worst consequences of the pandemic. This was accomplished in spite of the hurdles that were presented by early outbreaks, waves of infection, and the appearance of variants.

In order to effectively manage public health emergencies while maintaining a balance with the requirements of the economy, Switzerland’s experience highlights the need for adopting a flexible and responsive strategy.

Each country’s response was shaped by its healthcare system, government policies, and public compliance. Italy and Romania relied more on strict lockdowns, while Switzerland used a more balanced approach. Romania faced greater difficulties due to its underfunded healthcare infrastructure, while Italy and Switzerland managed resources more effectively, as seen in Table 1 [40,41,42].

Recent research has underscored the critical role of mathematical and statistical modeling in understanding and forecasting epidemic dynamics. Time-series methods such as the autoregressive integrated moving average (ARIMA) model have been widely employed to provide short-term forecasts of the COVID-19 spread, often with a promising accuracy [43,44]. While ARIMA models are effective in capturing temporal trends, they are limited in their ability to account for exogenous variables that strongly influence epidemic trajectories. To address this, researchers have employed ARMAX models, which incorporate external drivers such as population mobility, testing intensity, or policy interventions, thereby enhancing predictive robustness under heterogeneous conditions [45].

In parallel, the use of machine learning techniques, and neural networks in particular, has expanded significantly in epidemic modeling. Neural networks have proven effective in handling noisy, inconsistent, or incomplete epidemiological data while capturing nonlinear dependencies that are difficult to represent in traditional compartmental frameworks [46,47]. These data-driven methods complement classical epidemiological models by increasing flexibility and adaptability, especially in contexts where reliable data streams are scarce or fragmented. Collectively, this growing body of literature illustrates a shift toward hybrid approaches that combine statistical rigor with machine learning adaptability.

Building on this foundation, the present study advances the field in two key ways. First, it demonstrates that a predictive model originally developed on Romanian COVID-19 data can be successfully applied to Italy and Switzerland, validating its transferability across diverse epidemiological and socio-political contexts. Second, it enriches the modeling framework by incorporating exogenous variables, such as testing intensity, public health interventions, and imported cases into both neural network preprocessing and ARMAX modeling. These dual contributions to cross-country validation and enriched input modeling distinguish the study from prior work and offer a novel perspective on building resilient and transferable predictive frameworks for pandemic response. More broadly, the findings emphasize the value of integrating predictive analytics with resource allocation strategies to inform evidence-based decision-making and strengthen preparedness for future global health crises.

**Figure 1:**
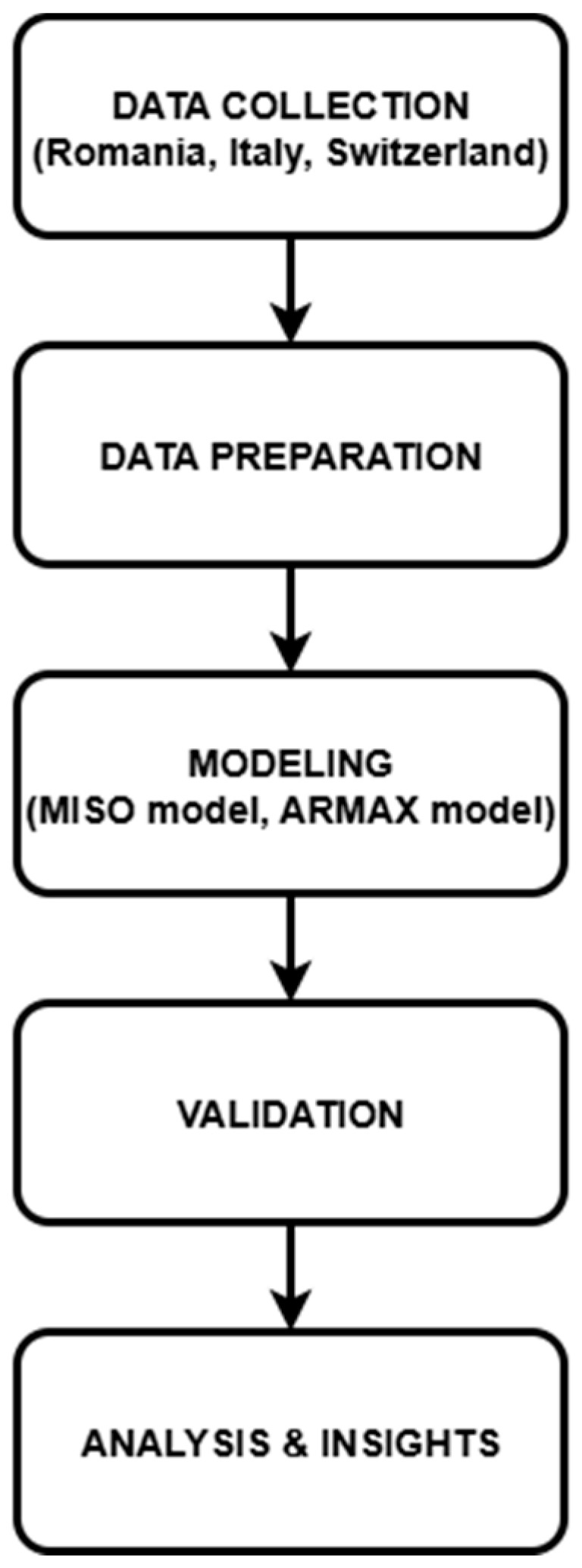
Study workflow diagram.

To provide a clear overview of the methodological approach, Figure 1 illustrates the sequential workflow adopted in this study. The process begins with data collection, followed by preprocessing, using the system such as MISO (Multi Input Single Output), mathematically modeling through ARMAX, validation across countries, and finally the generation of comparative insights.

The purpose of the first chapter is to serve as an introduction, which includes presenting a summary of the research and laying the groundwork for the subsequent analysis. The second chapter is devoted to the presentation of the data that were acquired for the purpose of model identification. Additionally, a comprehensive explanation of the mathematical modeling of the system is included in this chapter. In this section, we will explain the input signals that were employed in the modeling process, and we will also analyze the impact that these signals had on the spread of the COVID-19 virus. The fourth chapter includes a simulation of the suggested model, which is then followed by an explanation and interpretation of the findings of the simulation. It is also provided in this chapter that the analysis of the data is offered, which supports the validity of the model and its ability to foresee future events in the pandemic. In conclusion, the last chapter offers a synopsis of the results as well as insights into potential future research pathways that may be explored in this specific area of field of study.

A key contribution of this work is in demonstrating that a Romania-based predictive model can be successfully applied to Italy and Switzerland, thereby validating its transferability across heterogeneous epidemiological and policy contexts. The ability to extend a model calibrated on Romanian data to other European countries is particularly relevant given the marked differences in testing intensity, healthcare infrastructure, and timing of pandemic waves observed across these nations. By confirming that the model retains predictive accuracy beyond its initial development setting, the research provides strong evidence for the robustness of the proposed approach.

A further innovative dimension of this work is the explicit incorporation of exogenous variables into the modeling framework. Factors such as the intensity of testing, the range and timing of policy interventions, and the number of imported cases were all accounted for during the model training and validation phases.

Taken together, these dual contributions, a cross-country validation and the integration of exogenous drivers, offer a novel perspective on how pandemic dynamics can be analyzed in real time. Unlike traditional compartmental models that assume uniformity across populations, the present approach demonstrates adaptability and resilience under diverse epidemiological and socio-political conditions. More broadly, the findings underscore the potential of transferable, data-driven predictive models to inform evidence-based decision-making, support healthcare resource allocation, and enhance preparedness for future global health crises. By bridging methodological rigor with practical applicability, this study contributes to both the academic literature on epidemic modeling and the operational toolkit available to policymakers.

## 2. Data Processing and Mathematical Model Implementation

In order to provide an accurate assessment of the COVID-19 pandemic’s effects and patterns, data processing is an essential component. For the purpose of our research, we made use of validation data obtained from the World Health Organization (WHO) for three different countries: Switzerland, Romania, and Italy.

Processing and evaluating five major input data was the strategy that we used. These variables were the temperature inside, the temperature outside, the humidity, the number of tests that were carried out, and a quantified value that represented the actions that were taken by authorities to battle the pandemic.

Our approach, the tools we used to analyze the data, and the insights that we gained from the study are all detailed in this paper.

A complete dataset was made available by the World Health Organization (WHO) for the countries of Italy, Romania, and Switzerland. This dataset included historical and real-time data on COVID-19 cases, testing rates, and public health initiatives.

In order to supplement this dataset, we included information from other sources, such as meteorological records for temperature and humidity, as well as notifications from the government about limitations connected to the epidemic.

The major objective of this data-gathering endeavor was to determine whether or not there are any links between environmental parameters, testing rates, and the efficiency of control measures in regulating the spread of COVID-19.

When we started our research, preprocessing was an essential step that had to be taken to guarantee the dependability and precision of our data. During the preprocessing stage, the following processes were carried out:

- Preparing the Data: Imputation methods were used in order to successfully identify and rectify any missing information. By way of illustration, the values of temperature and humidity that were absent were filled in by utilizing interpolated meteorological data. To prevent the findings from being skewed, duplicate records were eliminated.
- Standardization and Normalization of the Data: As a result of the fact that our data included variables that were measured in multiple units (for example, temperature in Celsius, testing numbers as absolute counts). Additionally, standardization was carried out in order to guarantee consistency, especially with regard to the quantifiable values of official measurements, which presented a large degree of variation across the three nations.
- Utilizing Categorical Data Encoding: Measures taken by the government were first documented in the form of written descriptions. For example, we gave numerical values to these measurements by utilizing a preset scoring system that was dependent on the severity of the situation (for example, lockdown = 100%, social distance requirements = 50%, and no limits = 0%).
- The Engineering of Features: For the purpose of improving predictive analysis, new characteristics were developed. As an example, the rate of rise in testing was computed in order to gain an understanding of the responsiveness of testing procedures over the course of several years.

**Figure 2:**
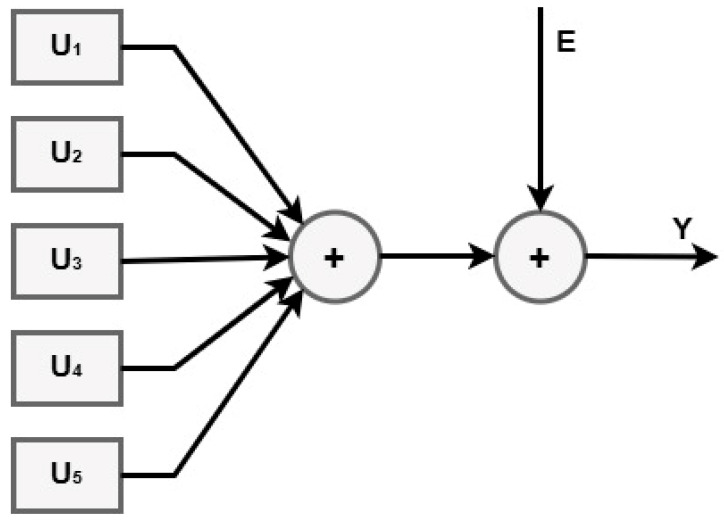
System design for mathematical modelling.

The designed system for the mathematical modeling of this process consists of five inputs and one output, which is basically translated into the MISO (Multi Input Single Output) model such as in Figure 2.

In this sense, an exploratory data analysis (EDA) was carried out with the primary emphasis being on the correlations between the variables. Among the most important discoveries were the following:

- Trends in Temperature and Case Analysis [48]: There was a correlation between lower temperatures outdoors and an increase in the number of COVID-19 cases in Italy and Romania, which suggests that seasonal influences may be at play. Because of Switzerland’s varied climate, the country displayed a variety of patterns, which necessitated a more in-depth examination on a regional level.
- Function of Humidity [48]: There was a modest decrease in the number of cases with higher humidity levels, which lends credence to the findings of the previous study that implies humidity may have an effect on the spread of viruses.
- Evaluation of the Effectiveness: A general correlation was found between an increase in the number of tests and an increase in the number of cases that were recognized [49]. This highlights the significance of extensive testing in the process of early detection and control.
- Impact of the Measures taken by the Government: It was shown that countries with tighter measurements (higher quantifiable values) had slower growth rates of infection, especially when paired with high testing rates.

Between the dates of 28 February and 29 October 2020, we took into consideration the COVID-19 case time-series data for three different nations: Romania, Italy, and Switzerland [50].

By using the latitude–longitude grid size, we were able to collect exposure data from the Copernicus ERA5 dataset. This allowed us to include the places that were stated. We chose the temperature and the temperature of the dew at a height of two meters above the surface, in addition to the surface downwelling shortwave radiation (solar UV radiation). The daily averages for these variables were obtained by selecting the grid cell that was geographically closest to each city or small area.

Through the use of the R “humidity” package (0.1.5.), we were able to determine the relative humidity (RH) by utilizing the temperature and the absolute humidity (AH).

The RH of the air was measured in relation to the concentration of water molecules at full saturation, whereas the AH of the air was measured in relation to the quantity of water vapor present in a certain volume of air.

The formula by which AH is related to relative humidity [51] and temperature is as follows (1):

(1) $$\mathrm{AH}=\frac{\mathrm{RH}\cdot e^{\frac{17.67\cdot T\left(℃\right)}{243.5+T\left(℃\right)}}\cdot 13.24}{273.15+T\left(℃\right)}$$

Regarding mathematical modelling, a study shows a selection framework that jointly considers predictive accuracy, time complexity, and scalability/runtime stability for operational public health forecasting, a step beyond accuracy-only comparisons [52]. Random Forest (RF) delivered the highest accuracy, but with greater temporal complexity and higher variance in prediction times (i.e., less predictable latency). XGBoost provided the best balance: strong accuracy, low prediction time variance, and good computational efficiency, so it is a recommended choice for real-time forecasting. Also, Support Vector Regression (SVR) was less accurate but the fastest (training and inference), making it attractive on resource-constrained hardware [52].

Another study builds on the six-compartment framework S, V, E, A, I, R described as follows: S(t) denotes the susceptibles; V(t) vaccinated individuals; E(t) those infected but not yet infectious (exposed); A(t) unreported infectious cases; I(t) infectious cases; and R(t) recovered individuals. The system is a non-autonomous compartmental model (humans and mosquitoes) with time-varying transmission/contact rates to capture seasonality; vaccination moves the susceptibles into a protected class and alters effective transmission to (partly) immune individuals. Fundamental properties, positivity of solutions, and a bounded invariant region were proved first [53]. On the other hand, there is a study that shows how combining multi-output Gaussian Processes (GP) with attention-based cross-series coupling can improve pandemic forecasting when signals are correlated across both what is predicted (cases vs. deaths) and where it is predicted (countries). Also, it proposes a Doubly Multi-Task Gaussian Process (DMTGP) that encodes task-wise correlations both across outcomes (cases–deaths) and across regions (country–country) so each time series borrows strength from the others. It adds a Transformer encoder to compute cross-attention among countries, capturing dynamic intercountry influence patterns that evolve over time. Attention maps qualitatively reveal these relationships [54].

Due to the fact that it takes into account external variables that have an effect on the number of cases, the ARMAX model is an effective instrument for forecasting COVID-19 instances.

According to ARMAX, the accuracy of COVID-19 case predictions may be improved in comparison to that of classic ARIMA models by the selection of important exogenous factors and the optimization of parameters.

Over the course of this research, the AutoRegressive Moving Average with eXogenous inputs (ARMAX) model was used in order to make a prediction regarding the quantity of COVID-19 instances.

Exogenous factors, such as environmental factors, testing rates, and government intervention measures, are included in the ARMAX model, which is an extension of the ARMA framework. The ARMAX (p, q, r) model may be expressed mathematically in (2):

(2) $$Y_{t}=c+\sum_{i=1}^{p}\varphi_{i}\cdot Y_{t-1}+\sum_{j=1}^{q}\theta_{j}\cdot \epsilon_{t-j}+\sum_{k=1}^{r}\beta_{k}\cdot U_{t-k}+\epsilon_{t}$$

The variable $Y_{t}$ reflects the number of daily COVID-19 instances, $X_{t}$ encompasses certain exogenous elements such as lockdown measures and number of tests taken, and $\epsilon_{t}$ is an error term that represents white noise.

The components of the ARMAX model are represented by the following:

- Autoregressive part (AR) described in (3):
  (3) $$Y_{t}=c+\varphi_{1}\cdot Y_{t-1}+\varphi_{2}\cdot Y_{t-2}+\cdots +\varphi_{p}\cdot Y_{t-p}+\epsilon_{t}$$
- Moving average part (MA) captures past predictions error (4):
  (4) $$Y_{t}=c+\theta_{1}\cdot \epsilon_{t-1}+\theta_{2}\cdot \epsilon_{t-2}+\cdots +\theta_{p}\cdot \epsilon_{t-p}+\epsilon_{t}$$
- Exogenous part (X) includes the external factors which affect the outcome (5):
  (5) $$Y_{t}=c+\beta_{1}\cdot X_{t-1}+\beta_{2}\cdot X_{t-2}+\cdots +\beta_{p}\cdot X_{t-p}+\epsilon_{t}$$

**Figure 3:**
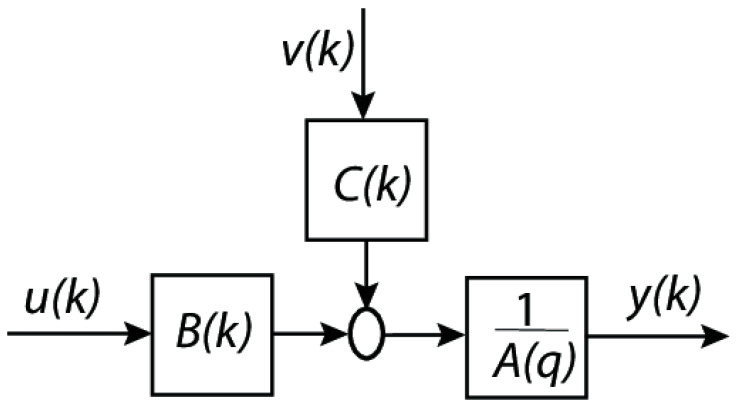
ARMAX model diagram.

A block diagram representation of a discrete-time ARMAX (AutoRegressive Moving Average with eXogenous inputs) model is shown in the diagram from Figure 3.

Components of the diagram are as follows:

- u(k): exogenous input signal (for example, an external control input or an external effect).
- Exogenous input filter, denoted by B(k), which simulates the way in which u(k) affects the system.
- Noise or disturbance (random noise or unmodeled effects that impact the system) is denoted by the variable v(k).
- C(k) is the moving average (MA) component, which describes the way in which noise from the past affects the system.
- By adding the contributions of B(k)u(k) and C(k)v(k), the summation block is completed.
- $\frac{1}{A\left(q\right)}$ is the autoregressive (AR) component (which represents the dynamics of the system).
- The output of the system (y(k)), which may be either the anticipated or observed value.

On the other hand, the mathematical representation of the system can be written like in (6):

(6) $$A\left(q\right)\cdot y\left(k\right)=B\left(q\right)\cdot u\left(k\right)+C\left(q\right)\cdot v\left(k\right)$$

where:

- A(q) is the autoregressive polynomial in the delay operator $q^{-1}$.
- B(q) is the exogenous input polynomial (how u(k) affects y(k)).
- C(q) is the moving average polynomial (how past noise affects y(k)).

**Figure 4:**
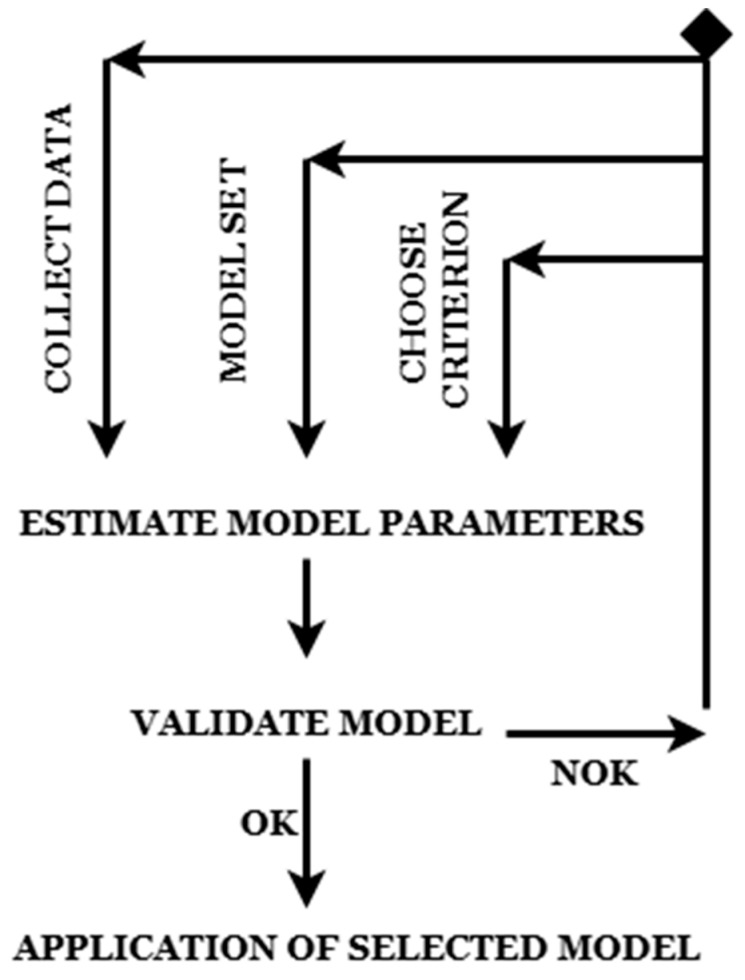
System identification diagram.

A data-driven method that is used in the process of developing mathematical models of dynamic systems is known as system identification (Figure 4). This approach is very important in the context of COVID-19 prediction since it helps to understand the transmission of the illness, forecast the number of cases, and evaluate the success of control measures. Through the utilization of data from the actual world, system identification provides researchers and policymakers with the ability to construct prediction models that bolster decision-making.

Collect Data: The first step involves gathering real-world data from the system to be modeled. In the case of COVID-19 prediction, this could include time-series data such as infection rates, mobility indices, vaccination levels, and lockdown measures. Data should be cleaned, preprocessed, and checked for stationarity before proceeding.

Model Set Selection: Different mathematical models can be considered, but in this case there is the ARMAX (AutoRegressive Moving Average with Exogenous Inputs).

Estimate Model Parameters: Model parameters are estimated using statistical techniques such as the maximum likelihood estimation (MLE) or the least squares estimation. In ARMAX models, this includes determining coefficients for AR (autoregressive), MA (moving average), and X (exogenous inputs) components.

Validate the Model: The estimated model is validated using a separate dataset or cross-validation methods. In this case, the comparison of predicted vs. actual values is used.

Decision Point: If the model performs well (OK), it can be applied for forecasting or system analysis. If the model fails (Not OK), adjustments are needed, which could involve:

Re-collecting data (if data quality is poor).
Once validated, the final model is deployed for real-world predictions.

In COVID-19 forecasting, this could mean predicting daily case numbers based on policy interventions and external conditions.

An approach that is organized and driven by data is provided by system identification, which is used for modeling and forecasting the spread of COVID-19. Through the integration of epidemiological information with contemporary methods of data analysis, it helps researchers and policymakers to make well-informed choices about the interventions and allocation of resources pertaining to public health.

## 3. Results

**Figure 5:**
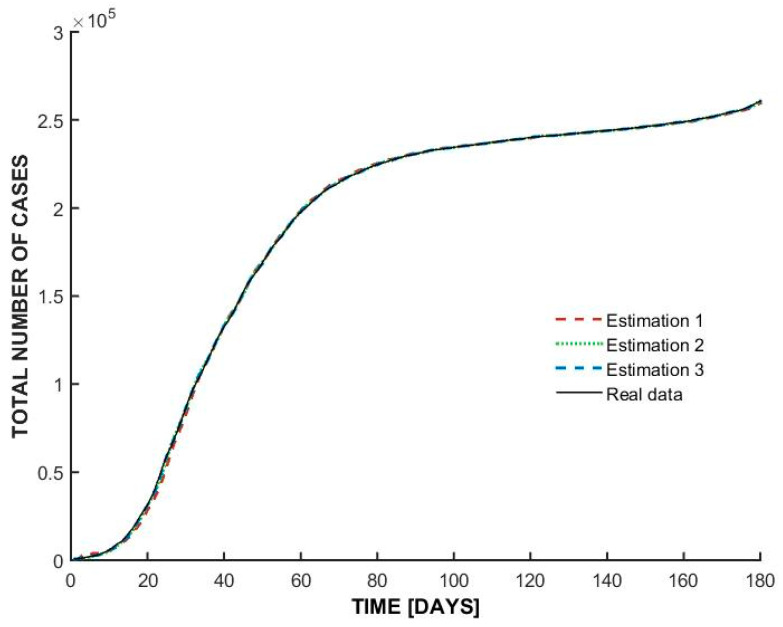
ARMAX model estimations for Italy.

The graph from Figure 5 represents the total number of COVID-19 cases in Italy over time (in days), comparing real data with three different estimations made by using ARMAX mathematical model.

The total number of cases follows an S-shaped (sigmoidal) curve, indicating an initial slow growth phase, followed by a rapid exponential increase, and then a plateau as cases stabilize. This is specific of an epidemic progression where the infection rate slows down as more people recover or become immune.

The three estimations presented in Table 2 (represented by dashed red, dotted green, and dashed blue lines) closely follow the real data (solid black line). This suggests that the models used for estimation are highly accurate in predicting the epidemic’s spread.

The small deviations between the estimations and real data indicate minor differences in model assumptions or parameter settings.

**Figure 6:**
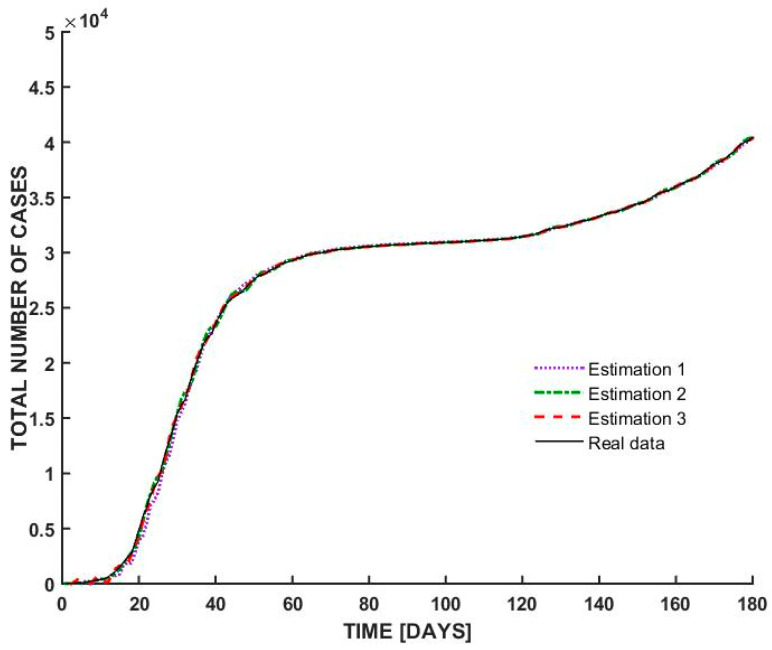
ARMAX model estimations for Switzerland.

On the other hand, the graph in Figure 6 shows the total number of COVID-19 cases in Switzerland over the same period.

The curve exhibits three distinct phases:

- At the beginning of the outbreak, the total number of cases increases sharply, indicating an exponential rise in infections. This suggests a highly contagious spread, typical of the early stages of a pandemic before interventions take effect.
- Around day 30, the curve begins to flatten, suggesting that the spread of the virus slowed down significantly. This could be attributed to government interventions such as lockdown measures, social distancing, and mask mandates. The plateau phase indicates that new infections were occurring at a lower rate, possibly due to public health measures successfully reducing transmission.
- After a prolonged period of stabilization, the curve starts rising again after day 100. This suggests a second wave or resurgence of cases, possibly due to relaxed restrictions, increased mobility, or changes in public behavior. Seasonal effects or new variants may have also played a role in driving up cases.

The three ARMAX-based estimations from Table 3 (dotted blue, dashed green, and dashed red lines) closely follow the real data (solid black line). This indicates that the ARMAX model effectively captures the real-world trend of COVID-19 case growth, making it a reliable tool for epidemiological forecasting.

Overall, the graph highlights the dynamic nature of pandemic trends and the effectiveness of mathematical models in tracking and predicting disease spread.

**Figure 7:**
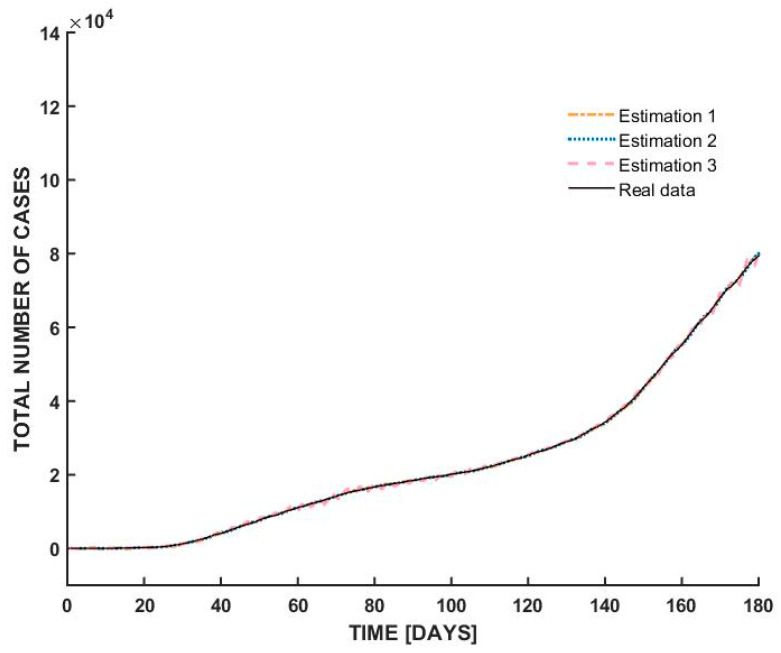
ARMAX model estimations for Romania.

The graph in Figure 7 illustrates the total number of COVID-19 cases in Romania over a 180-day period, comparing actual data with three separate estimations derived using the ARMAX model.

The highlighted feature of this graph is the remarkable alignment between the real data (black line) and the three model estimations (dashed lines). This convergence indicates that the ARMAX model—well-regarded for its capacity to incorporate both autoregressive components and external influences—has effectively captured the dynamics of the COVID-19 spread in Romania. All three estimations from Table 4 follow the empirical trajectory with minimal deviation, suggesting high accuracy and robustness in the model’s predictive capacity.

The graph shows a slow growth in the number of cases during the initial 40 days, likely reflecting early containment efforts. Around day 40, a noticeable inflection point appears, after which the total cases begin to rise more sharply. This phase shift may correspond to the relaxation of restrictions, increased transmission, or changes in testing capacity. From day 120 onward, the curve steepens significantly, indicating an accelerated spread, possibly due to the onset of a new wave or variant.

The relationship between the estimations and real data throughout all phases of the epidemic highlights the ARMAX model’s adaptability in capturing both linear trends and dynamic changes in disease progression. Such modeling is essential for policymakers, allowing them to simulate scenarios, evaluate interventions, and plan public health strategies based on reliable forecasts. Overall, the graph validates the efficacy of ARMAX modeling in real-time epidemiological forecasting.

The three graphs presented offer a comparative visualization of the total number of COVID-19 cases in Romania over a period of 180 days, each assessed using real data (solid black line) against three different estimations based on ARMAX models. While all graphs exhibit excellent alignment between real and estimated data, they represent different case trajectories, corresponding to the three countries.

Table 5 presents the quantitative performance metrics for the three graphs (the best fit obtained in each case). These results complement the visual alignment shown in Figure 5, Figure 6 and Figure 7 by providing numerical evidence of predictive accuracy.

## 4. Conclusions

The reaction of each nation was influenced by its healthcare system, the policies of the government, and the compliance of the general public. The countries of Italy and Romania relied more on lockdowns that were quite rigorous, while Switzerland took a more moderate approach. Romania saw additional challenges as a result of its inadequately financed healthcare system, while Italy and Switzerland were to a larger extent able to properly manage their resources.

This research aimed to test the application of a Romania-based prediction model for COVID-19 dynamics in Italy and Switzerland, with the twin objectives of evaluating its transferability and examining its relevance in informing resource allocation strategies. The results validate that the model maintained substantial predictive accuracy across several epidemiological settings, albeit considerable variations in testing intensity, intervention timing, and healthcare system capabilities.

The practical use of this modeling framework is in its possible application as a real-time decision-support instrument. By incorporating external variables such as testing rates and policy measures, the model can furnish policymakers with early warning indicators and scenario-based projections, facilitating more prompt interventions, optimized allocation of medical resources, and evidence-based modifications to public health strategies. Its capacity to generalize across many country settings underscores its value for regional and international collaboration amid forthcoming health emergencies.

Simultaneously, many limits must be recognized. The model’s accuracy is contingent upon the quality and consistency of the given data, which fluctuated across different nations and timeframes.

Furthermore, the simplifying assumptions inherent in the ARMAX and neural network frameworks may inadequately represent the intricacies of human behavior, virus mutations, or long-term socio-economic impacts. Policymakers could use this framework to anticipate infection surges, allocate ICU beds and ventilators more efficiently, and evaluate the likely impact of containment measures before implementation. However, its effectiveness depends on timely and consistent reporting of epidemiological data, and future improvements should aim to incorporate variant-specific dynamics and behavioral factors. Future research needs to concentrate on including more comprehensive information, expanding the model to include variant-driven dynamics, and improving its flexibility for real-time policy simulations.

This research illustrates the strength of a transportable predictive modeling framework and its ability to guide health policy and enhance pandemic preparation. The study integrates mathematical precision with practical use, offering both an academic advancement and a concrete resource for policymakers addressing swiftly changing public health crises.

As a result of our investigation, we came to the following essential realizations:

- Intervention by the government is absolutely necessary: in nations where the regulations were more stringent and were more effectively implemented, transfer rates were lower.
- There is no doubt that testing is an indispensable instrument: increased testing rates led to more precise case monitoring and improved overall management of outbreaks.
- There is a role played by environmental factors: there were observable and quantitative impacts of temperature and humidity on the propagation of the virus, which supported findings of seasonal trends.
- Policies that are driven by data are more effective: governments should make use of predictive models in order to make dynamic adjustments to their policies.

The lessons that may be learned from these experiences are very necessary in order to enhance future public health initiatives and to guarantee a more robust response to pandemics.

In the future, we may do the following to further enhance our analysis:

- Expanding the dataset to include new nations in order to validate it more thoroughly.
- Including other factors such as the population density, statistics on mobility, and vaccination rates in the analysis.
- Deep learning models are being used for the purpose of detecting more complicated patterns.

Current research’s predictive models serve as a reliable reference for optimal actions based on the pandemic’s progression. Nevertheless, in circumstances when financial, human, spatial, or temporal resources preclude adherence to these ideal methods, judgments will continue to be influenced by the human element, adept at assessing instances with the greatest likelihood of survival. The ethical challenge persists; yet, using predictive algorithms proactively yields valuable time in combating future infections. Organizations engaged in prompt decision-making during COVID-19 can publicly disclose extensive data on the recovery of severe and critical cases, utilizing criteria such as age, comorbidities, administered treatments, duration of hospitalization, and variations in recovery patterns. Consequently, mathematical models will markedly enhance predictions concerning optimal interventions in extreme triage scenarios and facilitate equitable allocation of healthcare resources.

The ethical implications of predictive models in facilitating triage and equitable allocation of healthcare resources need more exploration. By providing timely forecasts, models can help policymakers anticipate such bottlenecks, enabling pre-emptive measures such as mobilizing surge capacity, reallocating ventilators, or coordinating patient transfers across regions. Yet, the ethical dimension must not be overlooked: reliance on predictive tools to allocate scarce resources raises questions about fairness, transparency, and the risk of reinforcing existing inequalities. To mitigate these risks, predictive modeling should be embedded within broader ethical frameworks that ensure decisions respect human dignity and prioritize vulnerable populations.

We were able to provide a complete study of COVID-19 trends in Italy, Romania, and Switzerland by using powerful data processing methods. This research shed light on the interaction between environmental variables, testing, and governmental interventions. When it comes to the management of public health emergencies, this approach underscores the importance of data-driven decision-making.

## Figures and Tables

**Table 1:** Comparison of five most important factors between Italy, Switzerland, and Romania.

Factor	Italy	Switzerland	Romania
Healthcare Capacity	Overwhelmed, especially in Lombardy	Well-prepared, efficient ICU distribution	Underfunded, ICU shortages
Lockdown Measures	Strict nationwide lockdown	Partial restrictions, fewer lockdowns	Strict lockdowns but difficult enforcement
Testing and Tracing	Improved over time	Early adoption of digital tools	Limited due to resource constraints
Economic Support	Financial aid but deep economic hit	Strong financial packages for businesses	Struggled with economic relief
Vaccine Rollout	Rapid but faced some hesitancy	Well-organized and efficient	Slow due to hesitancy and misinformation

**Table 2:** ARMAX coefficients for Italy.

Estimation	Coefficients
Estimation 1	[2 2 2 1]
Estimation 2	[4 4 4 1]
Estimation 3	[3 3 3 1]

**Table 3:** ARMAX coefficients for Switzerland.

Estimation	Coefficients
Estimation 1	[2 2 2 1]
Estimation 2	[4 2 2 1]
Estimation 3	[6 4 4 1]

**Table 4:** ARMAX coefficients for Romania.

Estimation	Coefficients
Estimation 1	[2 2 2 1]
Estimation 2	[8 1 4 1]
Estimation 3	[6 1 2 1]

**Table 5:** Quantitative performance metrics.

Model	Best Fit Obtained
Italy	99.01%
Switzerland	98.67%
Romania	98.59%

## Data Availability

Data is contained within the article.

## Abbreviations

The following abbreviations are used in this manuscript:

MISO	Multiple Inputs Single Output
MIMO	Multiple Inputs Multiple Outputs
SISO	Single Input Single Output
WHO	World Health Organization
FFNN	Feed-Forward Neural Network
PID	Proportional Integral Derivative
PHEIC	Public Health Emergency of International Concern
